# Muscle-specific downregulation of GR levels inhibits adipogenesis in porcine intramuscular adipocyte tissue

**DOI:** 10.1038/s41598-017-00615-9

**Published:** 2017-03-30

**Authors:** Weiwei Chu, Wei Wei, Haiyin Han, Ying Gao, Kaiqing Liu, Ye Tian, Zaohang Jiang, Lifan Zhang, Jie Chen

**Affiliations:** 1 College of Animal Science and Technology, Nanjing Agricultural University, Nanjing, 210095 P.R. China; 2 Precision Medicine and Healthcare, Tsinghua-Berkeley Shenzhen Institute, Shenzhen, 518055 P.R. China; 3 College of Veterinary Medicine, Nanjing Agricultural University, Nanjing, 210095 P.R. China

## Abstract

Intramuscular adipose is conducive to good pork quality, whereas subcutaneous adipose is considered as waste in pig production. So uncovering the regulation differences between these two adiposes is helpful to tissue-specific control of fat deposition. In this study, we found the sensitivity to glucocorticoids (GCs) was lower in intramuscular adipocytes (IMA) compared with subcutaneous adipocytes (SA). Comparison of glucocorticoid receptor (GR) revealed that IMA had lower GR level which contributed to its reduced GCs sensitivity. Higher methylation levels of GR promotor 1-C and 1-H were detected in IMA compared with SA. GR expression decrease was also found in adipocytes when treated with muscle conditioned medium (MCM) *in vitro*, which resulted in significant inhibition of adipocytes proliferation and differentiation. Since abundant myostatin (MSTN) was detected in MCM by ELISA assay, we further investigated the effect of this myokine on adipocytes. MSTN treatment suppressed adipocytes GR expression, cell proliferation and differentiation, which mimicked the effects of MCM. The methylation levels of GR promotor 1-C and 1-H were also elevated after MSTN treatment. Our study reveals the role of GR in muscle fiber inhibition on intramuscular adipocytes, and identifies myostatin as a muscle-derived modulator for adipose GR level.

## Introduction

Adipose tissue is a large endocrine organ in the body, and functions in energy storage, hormone production, and immune function^1^. Most adipose tissues are deemed as waste in meat animal production, hence diminishing body fat content has long been an important goal of pig breeding. Consequently, subcutaneous adipose tissue (SAT), the major part of body fat, is supposed to be in the smallest amount possible in pig production. However, intramuscular adipose tissue (IMAT) is exceptionally regarded as favorite fat because it is conducive to pork quality. Moderate intramuscular fat content can promote pork juiciness, tenderness and flavor. Therefore, the breeding directions of IMAT and SAT are opposite, being positive for IMAT while negative for SAT. Unfortunately, reducing SAT always results in parallel decline of IMAT, which occurs prevalently in modern lean pig breeds. The reason might be that the regulation mechanisms for various fat tissues should be of similarities. Even they are from different fat depots. This explains why IMAT and SAT are of moderate positive correlation^2^.

However, adipose tissues from different locations also display their special physiological and biochemical characteristics. Many studies reported that subcutaneous adipocytes (SA) grew faster^3^ and accumulated more lipids^4^ than intramuscular adipocytes (IMA). Besides, the manners of their substrate utilization are different. The IMA consume more glucose, while SA mainly utilize exogenic fatty acids^5^. What’s more, differences of cell sizes^6^, secretory functions^7, 8^, levels of genes expression^9^, and hormone sensitivities^10, 11^ were observed between SA and IMA. Since the requirements for these two fat tissues are different, understanding the molecular regulation difference between IMAT and SAT is of great significance to pig breeding.

Fat deposition is a complex process involving proliferation and differentiation of adipocytes. Glucocorticoids (GCs) play vital roles in promoting fat deposition via inducing proliferation^12, 13^ and differentiation^14, 15^ of pre-adipocytes. And the adipocytes GCs sensitivity decides the ability of fat deposition. In human, visceral adipocytes (VA) are more sensitive to GCs than SA^16^. When the levels of GCs are induced, more lipids will deposit in VA, leading to “central obesity”^16–20^. However, the molecular mechanism underlying differential GCs sensitivities of various adipose tissues still remains unknown.

Basing on these findings, we hypothesize that the physiological differences between SA and IMA might be caused by the muscle specific regulation of adiposes. In current study, we investigate to learn whether GCs sensitivity is involved in the physiological differences between SA and IMA, and whether the local muscle-to-fat regulation of GRs is responsible for muscle-specific GCs sensitivity. Our results demonstrated a novel pathway for the adverse effects of skeletal muscle on adipocytes, which could contribute to specifically regulate IMA in pork quality improvement.

## Results

### IMA was less sensitive to GCs than SA

**Figure 1:**
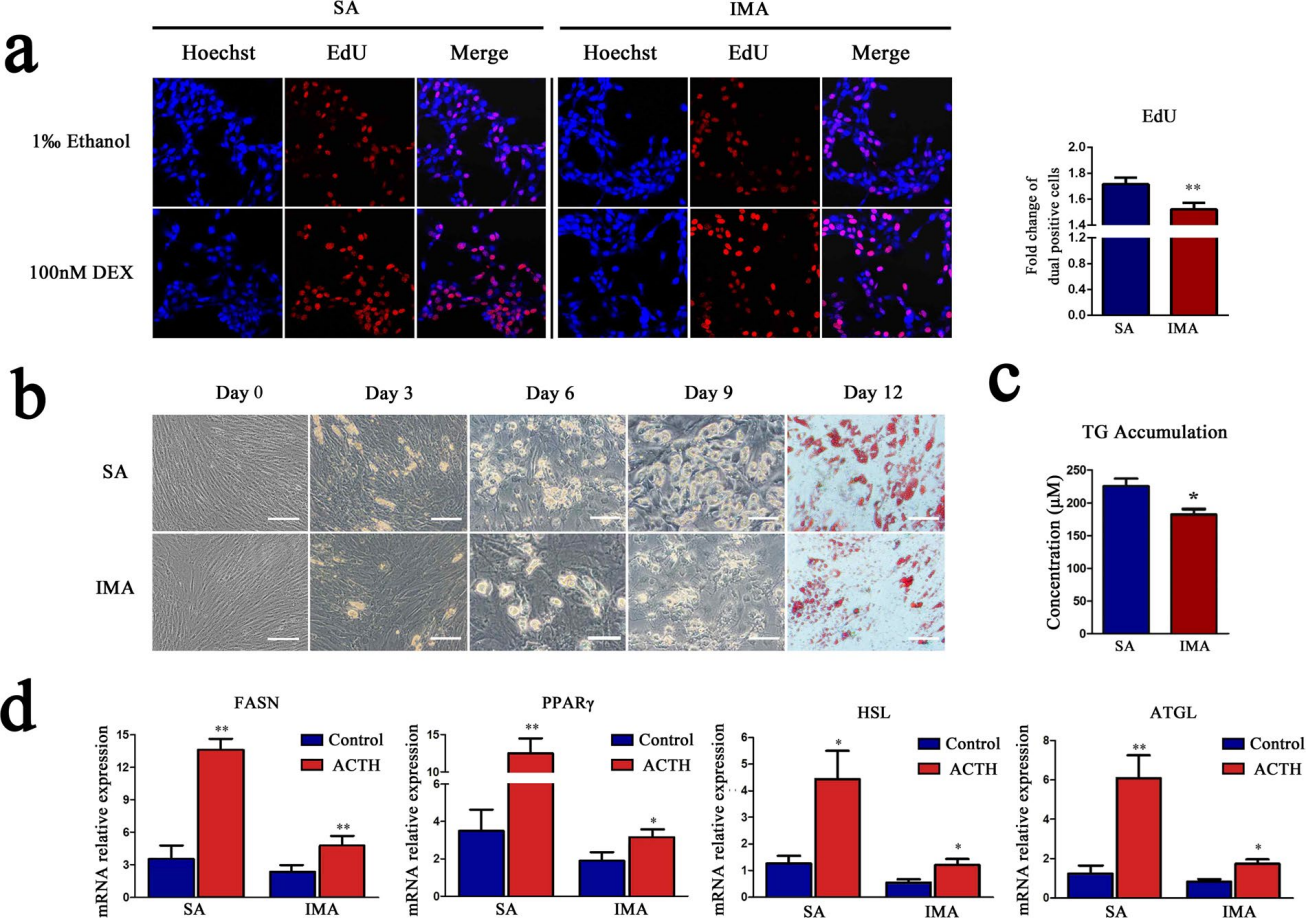
IMA was less sensitive to GCs than SA. (**a**) The EdU assay of SA and IMA pre-adipocytes. The porcine SA and IMA pre-adipocytes were treated with 1‰ ethanol or 100 nM DEX for 3 days, then the proliferating nuclei were stained red with EdU, the nuclei of all cells were stained blue with Hoechst for 2 hours. Three random pictures per group from confocal microscopy were used to count the cell numbers of EdU positive cells and Hoechst positive cells, and the ratio of the EdU positive cells to Hoechst positive cells was calculated in each picture. Data are shown as the mean ± SEM, n = 3 per group, *P < 0.05. (**b**) The differentiation of SA and IMA pre-adipocytes after induced with DEX. Cells were induced to differentiation by 2.5 μM DEX, and imaged by inverted microscope. Scale bar = 100 μm. (**c**) Triglyceride accumulation in SA and IMA treated as (**b**) was detected on day 9. Data are shown as the mean ± SEM, n = 3 per group, *P < 0.05. (**d**) mRNA expression of lipid metabolism genes in SA and IMA tissues of sows injected with 1 U/kg ACTH (n = 6) or equivalent volume saline (n = 6) intravenously per day for a total 9 days were detected. *Means significant differences between control and ACTH groups, *P < 0.05, **P < 0.01.

Many studies demonstrated that GCs played important roles in adipocytes proliferation^14, 15^ and differentiation^12, 13^. Adipose tissue with higher GCs sensitivity tends to deposit more lipids^10, 20^. However, the GCs sensitivity of IMA is still unknown. We compared GCs sensitivity between porcine IMA and SA *in vitro* and *in vivo*. SA and IMA pre-adipocytes were treated with 10, 100 and 1000 nM dexameson (DEX, GCs mimics) respectively. Cell viability assessment results showed that all three concentrations of DEX could promote the proliferation of SA and IMA pre-adipocytes, of which 100 nM DEX treatment is of the highest efficiency. When treated with 100 nM DEX for 24, 48 and 72 hours, IMA exhibited significantly lower viability than SA pre-adipocytes (*P* < 0.05) (Supplementary Fig. 1). After 100 nM DEX treated for 48 hours, EdU assay results indicated that the dual positive cells (proliferating cells) were significantly less in IMA than in SA pre-adipocytes (*P* < 0.01) (Fig. 1a).

DEX effect on the differentiation of SA and IMA pre-adipocytes was compared by treating pre-adipocytes with 2.5 μM DEX, which had been proved to be the optimal concentration for pre-adipocytes differentiation inducement^21^. On day 0 of treatment, SA and IMA pre-adipocytes were fibrous cells. During the process of differentiation, more and more lipid droplets were formed in both fat cells, but smaller and less lipid droplets were observed in IMA cells according to Oil Red O staining result (Fig. 1b). Results of triglyceride (TG) accumulation detection showed that TG concentration was also lower in IMA than in SA (*P* < 0.05) (Fig. 1c).

Adrenocorticotropic hormone (ACTH) stimulates the adrenal cortex to synthesize and secrete GCs. In this study, ACTH was injected intravenously for 9 days to compare GCs sensitivity of SA and IMA tissues *in vivo*. The result showed that administration of ACTH increased average plasma cortisol level by 2.2 folds (Supplementary Fig. 2). Fatty acid synthase (FASN) and peroxisome proliferator activated receptor γ (PPARγ) have key effects on the adipogenesis^22, 23^, and hormone sensitive lipase (HSL) and adipose triglyceride lipase (ATGL) are the maker genes of adipolysis^24, 25^. These genes were the downstream genes of the GR pathway, directly representing the status of the lipid metabolism. Here the elevated endogenous GCs significantly increased the expressions of these lipid metabolism genes in both SA and IMA tissues (*P* < 0.05) (Fig. 1d). More importantly, the mRNA expression fold changes of FASN [3.9(SA) vs. 2.1(IMA)], PPARγ [5.2(SA) vs. 2.2(IMA)], HSL [3.5(SA) vs. 2.0(IMA)], ATGL [5.2(SA) vs. 1.9(IMA)] were significantly less in IMA than in SA tissues (Supplementary Fig. 3).

### GRα was expressed less in IMA than SA

**Figure 2:**
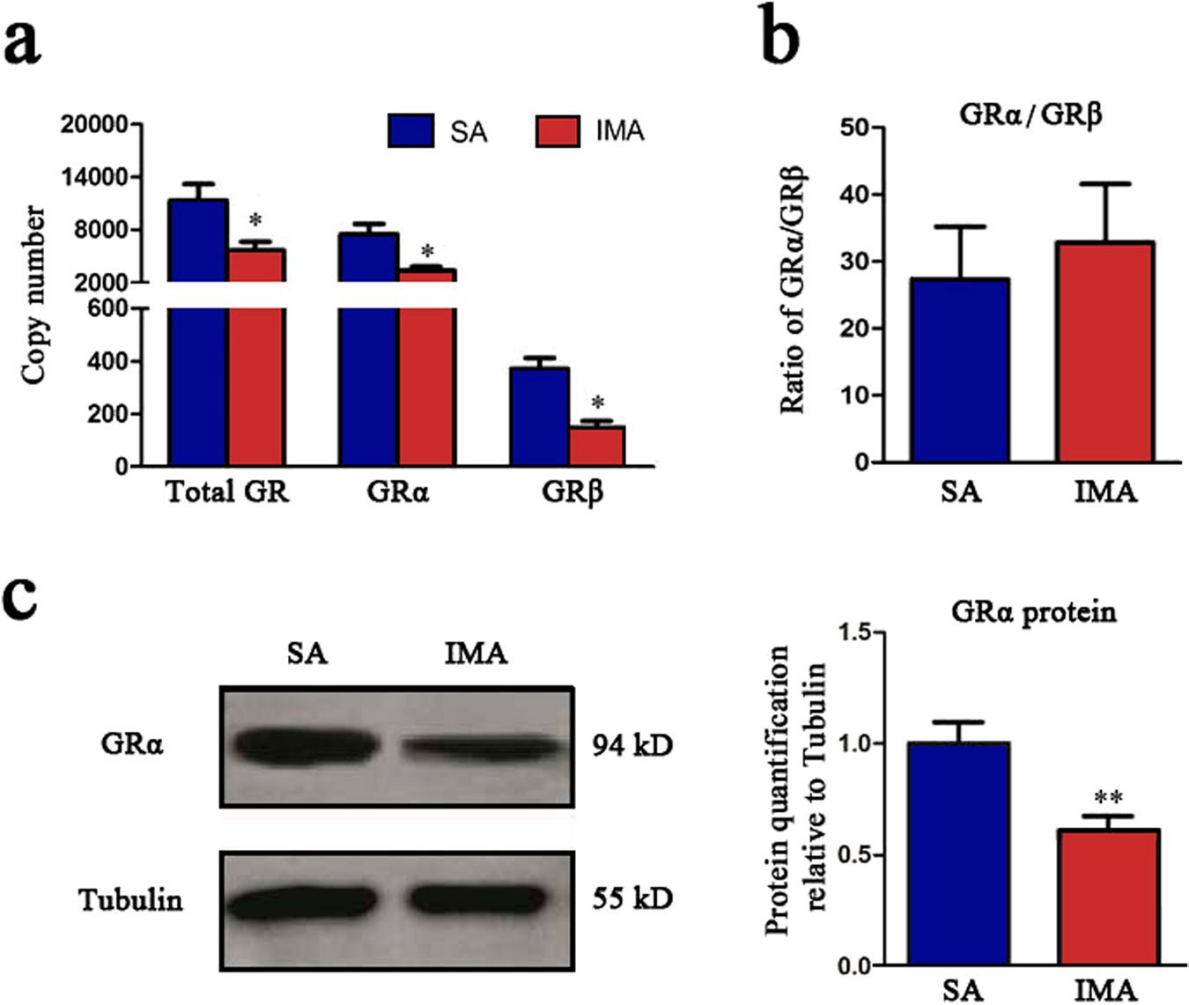
GR levels in IMA were lower than SA. Backfat and longissimus dorsi muscles were from 12 Erhualian sows, then the adipose tissues were collected for RNA and protein isolation. (**a**) Copy numbers of total GR, GRα, GRβ mRNA in SA and IMA tissues were detected by RT-qPCR. (**b**) The ratio of GRα/GRβ was displayed according to the result obtained in (**a**). Data are shown as the mean ± SEM, n = 12 per group, *P < 0.05. (**c**) Expression of GRα protein in SA and IMA tissues was detected by Western blot (left), and the relative protein expression level was displayed as column charts (right). n = 3 per group, **P < 0.01.

Circulating GCs can’t work as physiological factors without its receptor GR. GRα is the primary functional isoform of GR, while GRβ is the inhibitor of GRα. In order to reveal the mechanism of the different GCs sensitivities, we detected the GR expression levels of SA and IMA *in vivo*. SA and IMA tissues were obtained from 12 Erhualian pigs, and RT-qPCR results showed that total GR, GRα and GRβ mRNA were expressed less in IMA than in SA tissues (Fig. 2a), but the ratio of GRα/GRβ mRNA was no significant difference (Fig. 2b). The western blot analyses revealed that GRα protein was less in IMA than in SA tissues (Fig. 2c). These data indicated that GRα was the key factor for the differences of GCs sensitivities between SA and IMA.

### GRα levels were mainly determined by variants E1-C and E1-H

**Figure 3:**
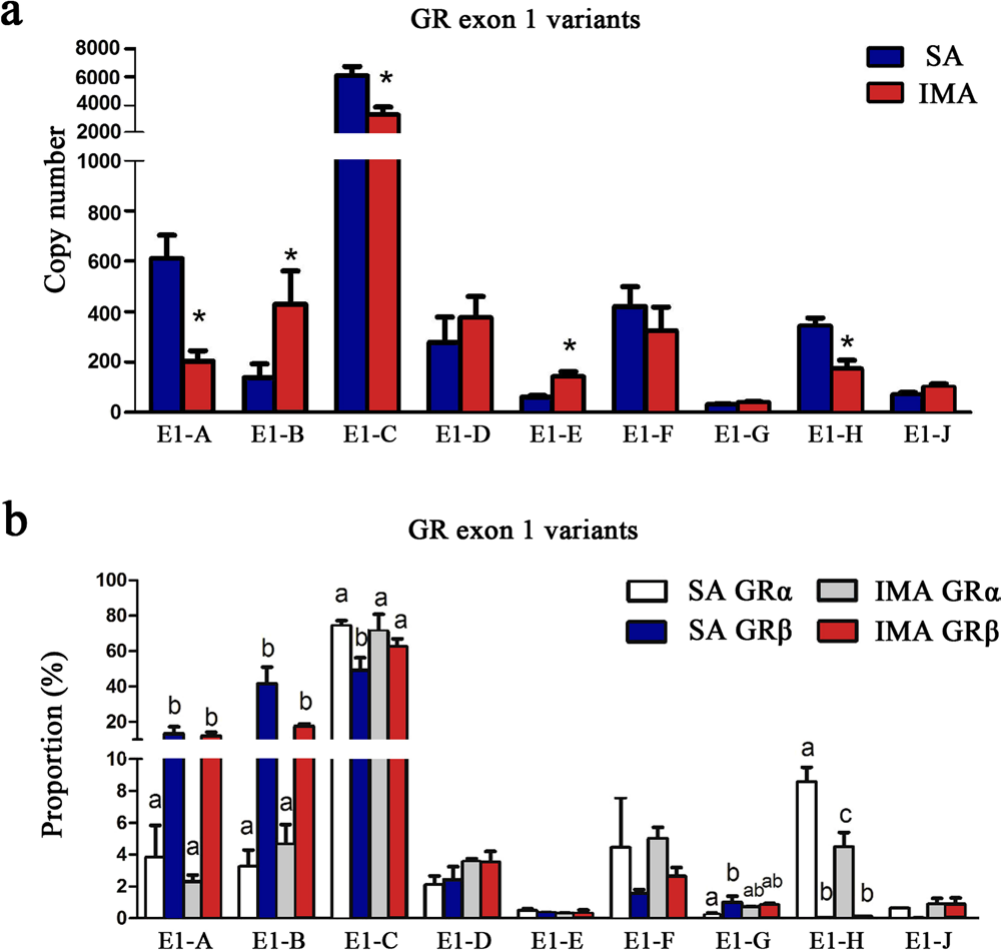
Expression patterns of GR exon 1 variants. (**a**) Copy number of GR exon 1 variants in SA and IMA tissues. n = 12 per group, *P < 0.05. (**b**) Variants proportion of GRα and GRβ in SA and IMA tissues. n = 6 per group, within column the different lowercases mean significant difference (One-way ANOVO, *P* < 0.05) within the columns, and the same lowercase means no significant difference (One-way ANOVO, *P* > 0.05).

The gene structure of GR is very complex, because the no-coding exon 1 contains different kinds of alternative splicing variants. To further identify which exon 1 variants mainly contribute to the different expression levels of GRα between SA and IMA, a total of nine types of exon1 variants were detected in SA and IMA tissues. The results showed that E1-C was the dominated type, accounting for 65.3~76.4% of the total GR mRNA. Thus, E1-C had a maximum effect on the total GR expression. Expression levels of E1-F, E1-D, E1-B, E1-A and E1-H were medium (1.6~10.2%), and the expression level of E1-E, E1-J and E1-G were slight (0.4~2.8%). Notably, E1-A, E1-C and E1-H were less expressed in IMA than SA tissues (Fig. 3a). For GRα and GRβ, the expression patterns of nine variants were not the same. Expressions of E1-A and E1-B were significantly higher in GRβ than in GRα, indicating that E1-A and E1-B tend to have more effects on the GRβ expression. Whereas E1-H was detected only in GRα, suggesting that E1-H transcript only encodes GRα in SA and IMA tissues. Moreover, E1-H level in SA was 1.9 times as high as that in IMA tissues (Fig. 3b). Therefore, higher levels of GRα in SA were mainly due to the higher levels of E1-C (the dominated variant) and E1-H (GRα specific variant).

### Muscle conditioned medium suppressed adipocytes proliferation and differentiation by reducing GRα expression

**Figure 4:**
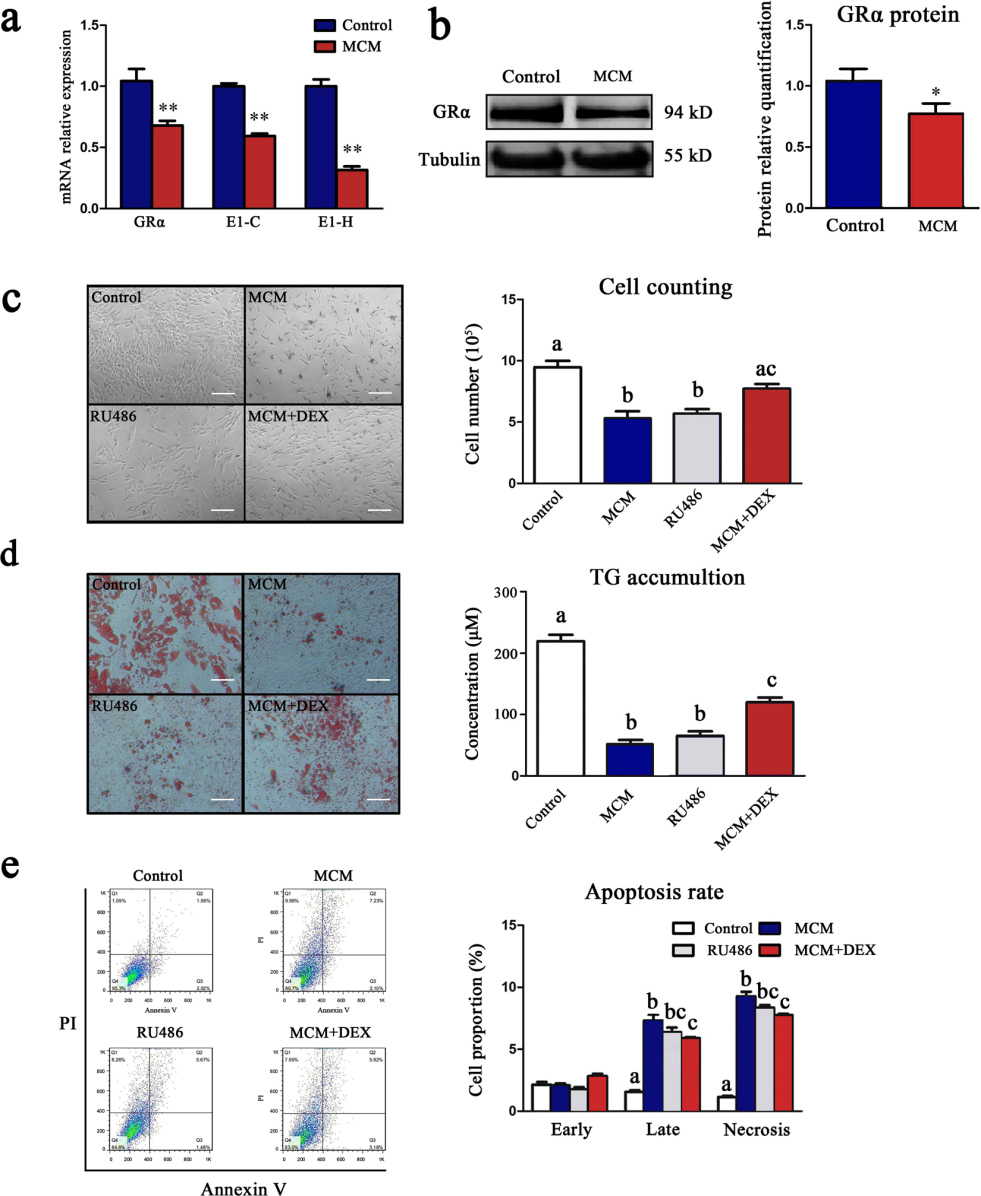
Skeletal muscle conditioned medium inhibited the adipogenesis by reducing GRα expression. The subcutaneous pre-adipocytes were cultured with skeletal muscle conditioned medium (MCM) or ordinary medium for 3 days, then the cells were collected for RNA, protein isolation and flow cytometry assay. (**a**) The mRNA expression of GRα and E1-C, E1-H was detected by RT-qPCR, n = 6 per group. (**b**) Expression of GRα protein was detected by Western blot (left) and displayed as column charts after quantification (right), n = 3 per group, *P < 0.05. (**c**) The effects of MCM on the proliferation of pre-adipocytes were detected by cell imaging (left) and cell counting (right). Cells were cultured with normal culture medium, MCM medium, normal culture medium with 100 nM RU486, or 100 nM DEX + MCM medium respectively, n = 6 per group. The different lowercases mean significant difference (One-way ANOVO, *P* < 0.05) within the columns, and the same lowercase means no significant difference (One-way ANOVO, *P* > 0.05), the same below. (**d**) The effects of MCM on the pre-adipocytes differentiation. After reaching confluence, pre-adipocytes were induced to differentiation for 12 days, then the Oil-red O staining (left) and TG assay (right) were conducted. n = 3 per group. (**e**) The effects of MCM on the apoptosis of pre-adipocytes were detected by flow cytometry (left) and displayed as column charts after quantification (right), n = 3 per group.

From the above results we concluded that the less expression of GRα variants E1-C and E1-H in IMA mainly cause its lower sensitivity of GCs compared with SA. Considering its special location, we assumed that the GRα levels in IMA were influenced by muscles. In order to mimic the effects of muscle fibers on normal adipocytes (haven’t been influenced by muscle), we used skeletal muscle conditioned medium (MCM) to treat subcutaneous pre-adipocytes. The results showed that the levels of variants E1-C and E1-H were reduced by 32.6% and 73.3% respectively, and GRα level was reduced by 46.4% on day 3 of treatment (Fig. 4a,b). Cell counting revealed that the cells number in MCM group was decreased by 41% compared with the control group. Treating pre-adipocytes with 100 nM RU486, a GR antagonist, had similar inhibitory effects, as the number of cells decreased by 38%. Notably, 100 nM DEX could rescue the inhibitory effects of MCM on the pre-adipocytes proliferation, as the cells number in MCM + DEX group was significantly larger than MCM group (*P* < 0.05) (Fig. 4c). On day 12 of inducing differentiation, Oil red O staining displayed that MCM inhibited the differentiation of pre-adipocytes. TG assay showed that the TG concentration decreased by 45% (Fig. 4d). Moreover, the late apoptosis rate of pre-adipocytes increased 3.3-fold, and cell necrosis rate increased 8.0-fold, which indicated that MCM promoted the apoptosis of pre-adipocytes. RU486 had a similar effect on pre-adipocytes apoptosis, which increased late apoptosis rate by 2.4-fold, and cell necrosis rate by 6.8-fold. Adding DEX to MCM could rescue the effects of MCM on the pre-adipocytes apoptosis (Fig. 4e).

### Myostatin inhibited adipogenesis and reduced GRα expression in adipocytes

**Figure 5:**
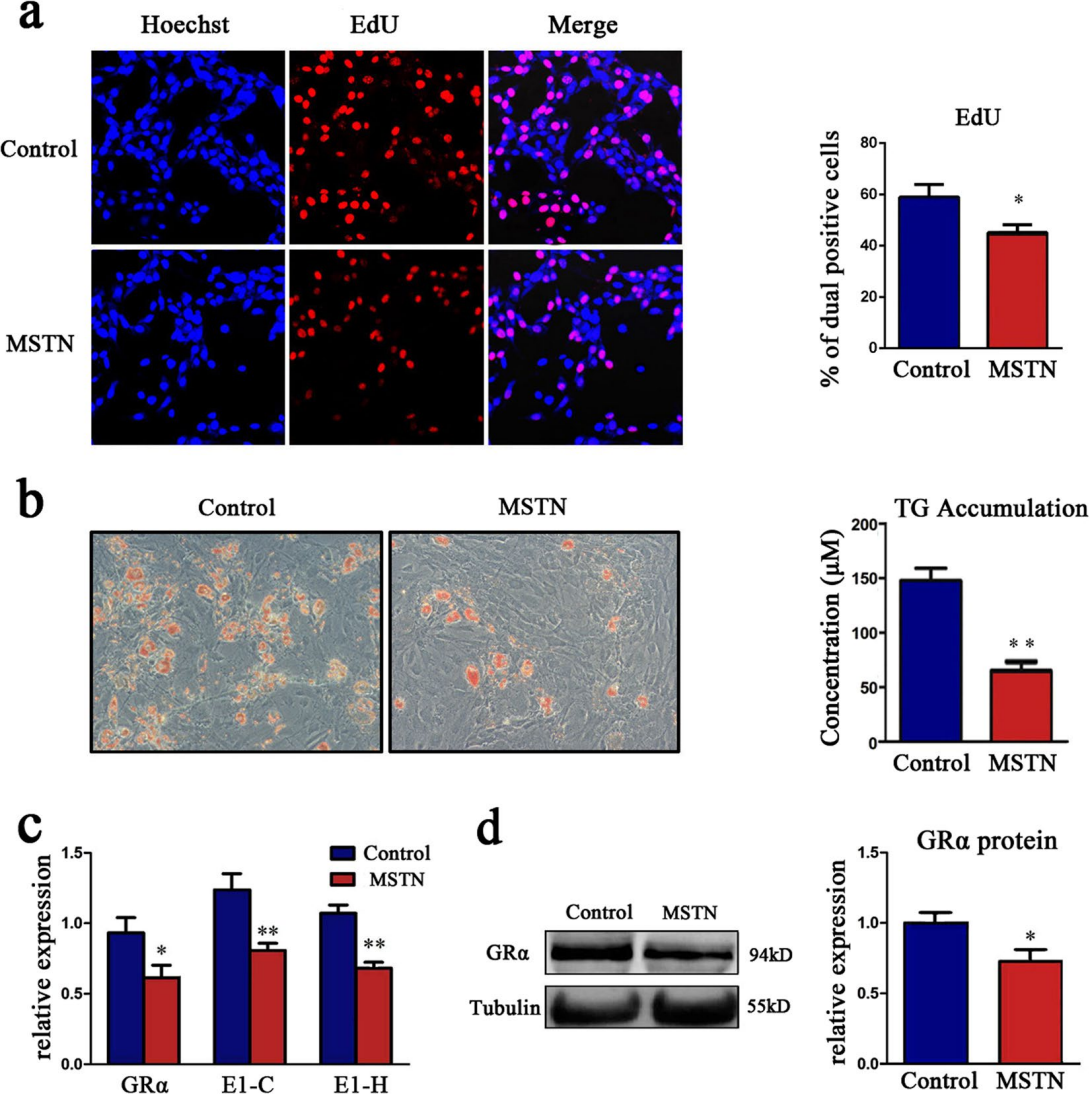
MSTN inhibited adipogenesis and reduced GRα expression. (**a**) The effects of MSTN on the proliferation of subcutaneous pre-adipocytes. Pre-adipocytes were treated with 50 ng/ml MSTN for 2 days, then the proliferating nuclei were stained red with EdU for 2 hours, and nuclei of all cells were stained blue with Hoechst (left). Three random pictures per group from confocal microscopy were used to count the cell numbers of EdU positive cells and Hoechst positive cells, and the ratio of the EdU positive cells to Hoechst positive cells was calculated in each picture (right). (**b**) Oil red O staining (left) and TG assay (right), n = 3 per group. The pre-adipocytes were cultured in culture medium until full confluence, then the cells were induced to differentiation with PBS or 50 ng/ml MSTN for 9 days. (**c**) The mRNA expression of GRα, exon 1C and 1H was detected by RT-qPCR, n = 6 per group. (**d**) The protein expression of GRα was detected by Western blot (left) and displayed as column charts after quantification (right), n = 3 per group. *P < 0.05; **P < 0.01.

Myostatin (MSTN) is a special myokine secreted by muscle. High concentration of MSTN was also detected in MCM in this study (Supplementary Fig. 4). We previously found that MSTN could inhibit the differentiation of pre-adipocytes^26^. Therefore, we presumed that MSTN contributes to the MCM inhibiting effect on intramuscular adipocytes. Firstly, we assessed the effect of MSTN on adipogenesis. After treating subcutaneous pre-adipocytes with 50 ng/ml MSTN for 2 days, we found the proportion of EdU positive cells decreased by 31% (Fig. 5a), indicating that MSTN could suppress pre-adipocytes proliferation. To detect the effects of MSTN on differentiation of pre-adipocytes, the pre-adipocytes were cultured in culture medium until full confluence, and then the cells were induced differentiation with or without 50 ng/ml MSTN for 9 days. Oil red O staining and TG assay revealed that MSTN significantly inhibited pre-adipocytes differentiation, as the TG concentration in adipocytes decreased 46% after MSTN treatment (Fig. 5b).

We further aim to detect whether MSTN play a role in the muscle fiber inhibition on IMA GR expression. After treated with 50 ng/ml MSTN for 3 days, the mRNA levels of GRα, E1-C and E1-H in subcutaneous pre-adipocytes were reduced by 35.8%, 34.5% and 33.4% respectively (Fig. 5c), and the levels of GRα protein decreased by 24% compared with the control group (Fig. 5d).

### Myostatin influenced the cell cycles and apoptosis of adipocytes

**Figure 6:**
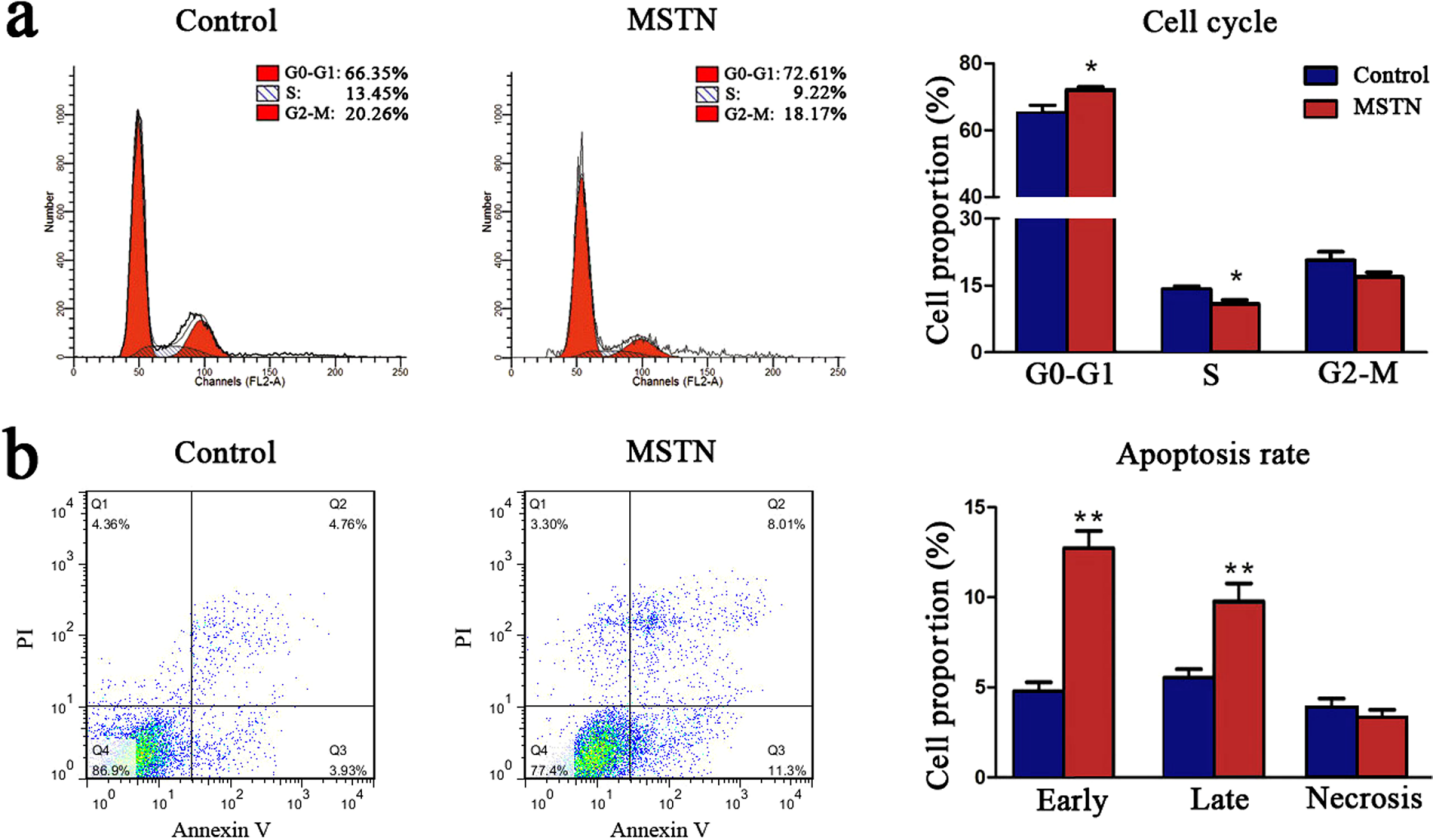
MSTN influenced cell cycles and apoptosis of adipocytes. Subcutaneous pre-adipocytes were treated with 50 ng/ml MSTN for 2 days and then harvested for further detection. (**a**) The effects of MSTN on the cell cycles of pre-adipocytes. Cells were stained with PI, then the cell cycles were detected by flow cytometry (left) and subsequently displayed as column charts after quantification (right), n = 3 per group. (**b**) The effects of MSTN on the apoptosis of pre-adipocytes. Cells were stained with PI and Annexin V, then the apoptosis rate was detected by flow cytometry (left) and displayed as column charts after quantification (right). n = 3 per group, *P < 0.05; **P < 0.01.

After treated with MSTN for 2 days, Flow cytometry was used to detect the cell cycles and apoptosis of pre-adipocytes. The results showed that proportion of subcutaneous pre-adipocytes in G0-G1 phase was increased by 8.3%, and cells in S phase decreased by 5.1%, suggesting that MSTN arrested pre-adipocytes in the G0-G1 phase (Fig. 6a). Besides, early and late apoptosis rates were increased by 220% and 90% respectively (Fig. 6b), indicating the promotion role of MSTN for pre-adipocytes apoptosis.

### Higher methylation levels of GR promoter 1-C and 1-H were detected in IMA

**Figure 7:**
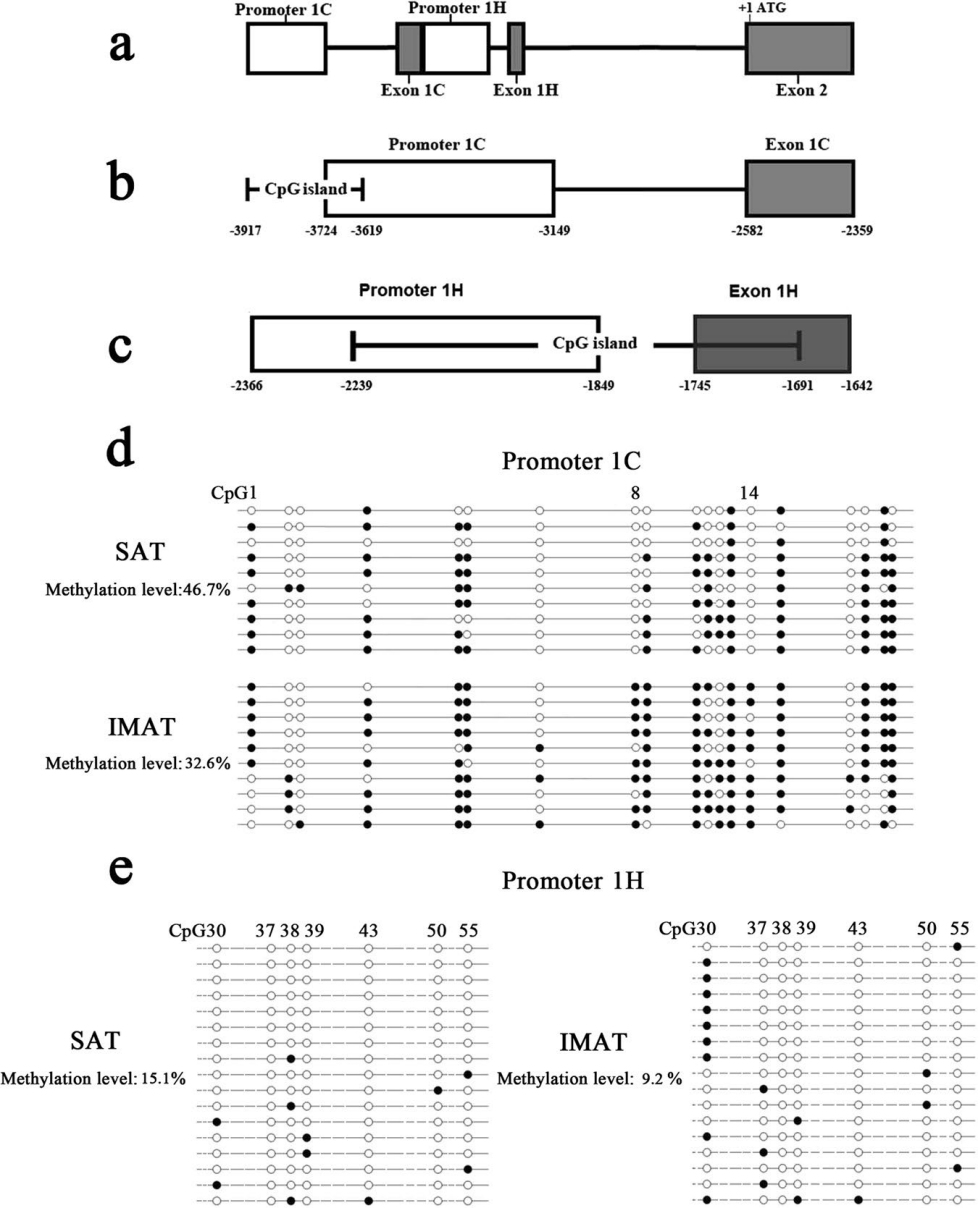
Methylation levels of promoter 1-C and 1-H were higher in IMA. (**a**) Locations of promoter 1-C and 1-H. The gray vertical bars represent the exons of GR gene; the open bar represents the promoter. (**b**) Location of CpG island in promoter 1-C. (**c**) Location of CpG islands in promoter 1-H. Nucleotide numbering is relative to + 1 at the initiating ATG codon. (**d**) Methylation status of promoter 1-C in IMA and SA. (**e**) Methylation status of promoter 1-H in IMA and SA. Each line represents an individual bacterial clone that was sequenced. Open circles indicate unmethylated CpG sites. Black circles indicate methylated CpG sites.

DNA methylation modification is one of important ways to regulate GR expression^27, 28^, and thus we analyzed the methylation levels of GR promoter in SA and IMA tissues. The locations of variant E1-C and E1-H promoters were showed in Fig. 7a–c, and the CpG sites were named CpG1~55 according to their orders in sequence. Bisulfite sequencing results showed that the methylation level of promoter 1-C was 46.7% in IMA pre-adipocytes, which was significantly higher than that in SA pre-adipocytes (32.6%) (*P* < 0.05) (Fig. 7d). For promoter 1-H, the methylation level in IMA (15.1%) was significantly higher than that in SA pre-adipocytes (9.2%) (*P* < 0.05) (Fig. 7e).

### Myostatin elevated the methylation levels of promoter 1-C and 1-H

**Figure 8:**
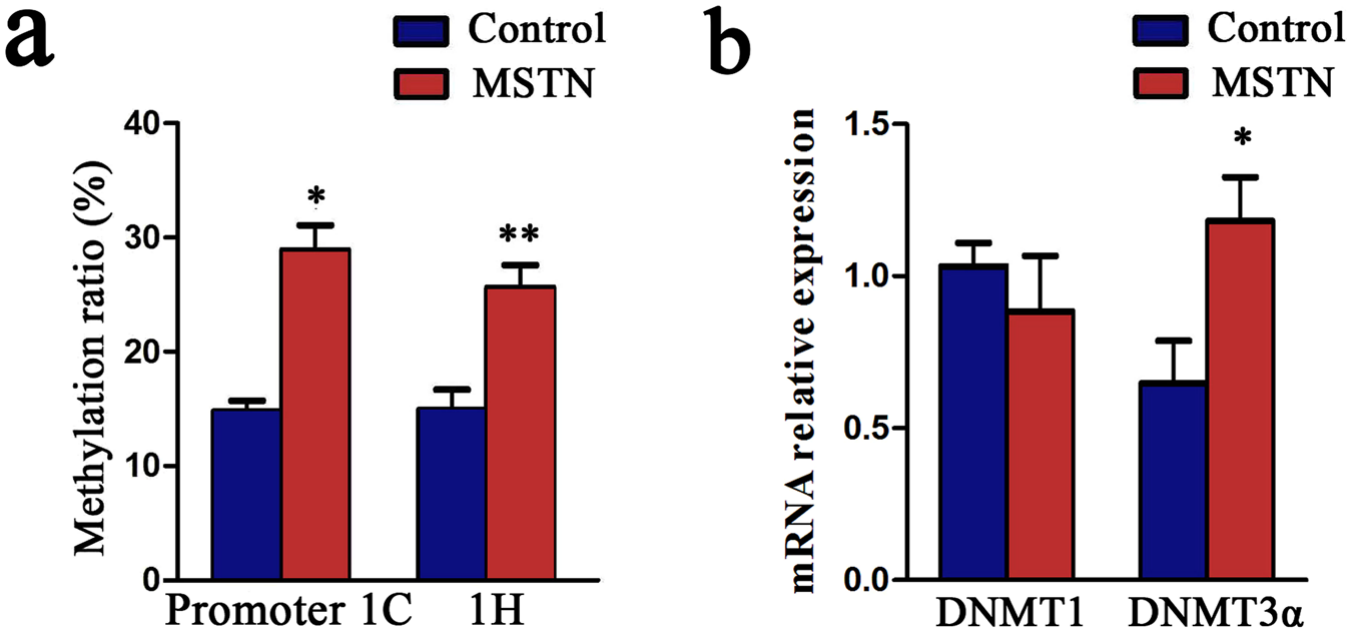
MSTN elevated the methylation levels of promoter 1-C and 1-H. After treated with 50 ng/ml MSTN for 2 days, the pre-adipocytes were harvested, then the methylation status of promoter 1-C and 1-H were detected by methylated DNA immunoprecipitation method (**a**), and the relative mRNA expression of DNA methyltransferase DNMT1 and DNMT3α were detected by RT-qPCR (**b**). n = 6 per group. *P < 0.05; **P < 0.01.

We further detected whether MSTN influence methylation levels of GR promoter in pre-adipocytes by DNA methylation immunization (MeDIP). After treated by 50 ng/ml MSTN for 2 days, the methylation levels of promoter 1-C increased from 15% to 29%, and the methylation levels of promoter 1-H increased from 15% to 26% (Fig. 8a). RT-qPCR results demonstrated that DNMT3α was upregulated when treated by MSTN (Fig. 8b).

## Discussion

Distribution of body fat is an important predictor of metabolic abnormalities, because subcutaneous adipose is inversely associated with glucose and lipid concentrations, while increased intramuscular adipose is independently associated with insulin resistance and T2D^29, 30^. Previous studies indicated that the proliferation and differentiation abilities, levels of genes involved in lipid metabolism were dramatically lower in IMA than SA^7, 9, 31, 32^. Unclosing the mechanism of the lower ability of adipogenesis in IMA is of great medical and economic value. As we know, glucocorticoids (GCs) is a key factor to regulate the proliferation^12, 13^ and differentiation^14, 15^ of adipocytes. Adipose with higher GCs sensitivity tend to deposit more lipids^10, 20^. For example, the visceral adipose is more sensitive to GCs than subcutaneous adipose^16^, and when the levels of GCs were elevated, more lipids would deposit in visceral adipocytes, leading to “central obesity”^17–19^. In present study, we tend to uncover why the lipids prone to deposit less in IMA, which could be helpful to regulating IMA specially in pork quality improvement. Our results indicated that the GCs induced-proliferation, differentiation and the marker genes’ expression were lower in IMA compared with SA, that lead to less lipids were deposited in IMA.

Physiological effects of circulating GCs are mediated by the GR. So it is reasonable that the levels of GR influence GCs sensitivity, and it is confirmed by lots of studies. Cortisol resistance is accompanied by a reduction of GR^33, 34^, however high levels of GR increased GCs sensitivity^35^. GRα is a hormone-activated transcription factor, while GRβ is transcriptionally inactive and is a potential inhibitor of activated GRα^36^. The ratio of GRα/GRβ usually influences the GCs sensitivity. In present study, lower levels of GR mRNA and protein were detected in IMA, but the ratio of GRα/GRβ was no difference between IMA and SA. Thus, so we only took GRα as our target in next study.

What’s more, the isoforms of the GR were influenced by the expression of alternative exon 1 variants in different species and tissues^37–40^. To uncover the primary exon 1 variants in dominating the tissue specific expression of GRα, we measured the express patterns of nine GR variants in SA and IMA. The results showed that E1-C was the highest expressed variants, and expressions of E1-A, E1-C and E1-H were significantly lower in IMA. Further study on the express patterns of variants for GRα and GRβ showed that E1-A mainly existed in GRβ other than GRα. Notably, E1-H was only detected in GRα, which means the E1-H does not contribute to GRβ expression. Therefore, the lower level of GRα in IMA was mainly caused by the lower expression of E1-C (the primary variant) and E1-H (GRα specific variant). Previous reports disclosed that E1-C account for a proportion of 70% to 80% in different porcine tissues^40, 41^, and the mini genes which contain E1-H increased the proportion of GRα^42^, which is coincident with our present results.

Most studies of muscle-adipose crosstalk were focused on the effects of adipose on the muscle, only a few studies reported that myocytes suppressed differentiation of adipocytes^43, 44^, and degenerating myofibres could affected the cellular morphology of 3T3-L1 preadipocytes^45^. However, the mechanism was still unknown. Considering the special location of IMA, we assumed GRα level in IMA influenced by muscles. We treated porcine adipocytes with MCM to mimic the effects of muscle on the adipocytes *in vitro*, finding that the expression of E1-C, E1-H and GRα was inhibited, which confirmed our hypothesis. Along with the reduction of GRα, the proliferation and differentiation of adipocytes were also suppressed, and the apoptosis was induced. The similar effects were observed when adipocytes were treated with RU-486 (GR antagonist), and DEX could partly recover the negative effects of MCM. These data coincides with the previous studies, and unclosed that MCM inhibited adipogenesis by reducing GR.

In our previous study, we co-cultured 3T3-L1 pre-adipocytes with C2C12 myotubes by trans-well inserts, and found that C2C12 cells inhibited the proliferation and differentiation of 3T3-L1 cells^46^. Muscle has been identified as an endocrine organ owing to its capacity to produce and secrete a variety of myokines and other proteins^47^. Some cytokines including IL-1α, IL-2, IL-4, TNFα, INFα and NF-κB could impact the GR expression in many cell types^48–50^. Besides, the other myokines including IL-6, IL-15, TNFα, Irisin, and MSTN which could inducing thermogenesis or inhibit adipogenesis might have the ability to regulate expression of GR^26, 51–54^. Our previous study found that myokines MSTN inhibited the differentiation of porcine adipocytes^26^. Hence, we detected the concentration of MSTN in muscle, and the results showed that concentration of MSTN in muscle MCM was extremely high. Next, when we treated adipocytes with MSTN, the GRα level was significantly reduced, in accord with the effects of MCM. MSTN also inhibited the proliferation and differentiation of porcine adipocytes, which confirmed our previous results.

DNA methylation is an important epigenetic modification mechanism and has traditionally been associated with gene repression^55^. Lots of studies showed that GR expression was suppressed by high levels of methylation^27, 28^. What’ more, MSTN was reported to have the ability to regulate DNA methylation levels in muscle satellite cells^56^. By bisulfite sequencing, we detected higher methylation levels of promoter 1-C and 1-H in IMA than SA, and these results were in accordance with the express pattern of exon 1-C and 1-H. Hence, having treated adipocytes with MSTN, we found that the main de novo methyltransferases DNMT3α^57^ was induced, and the methylation levels of promoter 1-C and 1-H were increased.

MSTN is predominantly expressed in muscle, and was usually considered as a negative regulator of muscle growth^58^. Recently, many reports found that MSTN also blocked the differentiation of adipocytes^59, 60^. As a member of TGF-β superfamily, MSTN works by binding to activine type II receptor. Then the Smad family members are subsequently activated and form a complex, which are then translocated to the nucleus, regulating target gene transcription^61^. The cooperation between Smads and AP1^62^, KLF5^63^ has been reported in previous studies, and the binding sites of these two important transcription factors were found in the promoter of DNMT3α. Thus MSTN probably regulate DNMT3a through the cooperation between Smads and these transcription factors.

In summary, we found that muscle secreted MSTN regulated the methylation levels of promoter 1-C and 1-H in adipocytes, leading to the reduction of GRα level. This effect lowered the GCs sensitivity of IMA, and decreased the GCs-induced proliferation, differentiation and anti-apoptosis of adipocytes, therefore reducing fat deposition in IMA. This study provides a novel molecular mechanism of how the local myocytes inhibits adipocytes, and could contribute to specifically regulate IMA without influencing the other fat tissues deposition.

## Methods

### Ethics statement

All experiments were performed in accordance with the guidelines of the regional Animal Ethics Committee and were approved by the Institutional Animal Care and Use Committee of Nanjing Agricultural University (NJAU-CAST-2014-179).

### Cell isolation and culture

All the animals used in this study were treated in accordance with “The Regulations on the Care for Laboratory Animals” of the Ministry of Science and Technology of the People’s Republic of China. Three-day-old female Erhualian pigs were killed via intraperitoneal injection of pentobarbital sodium (50 mg/kg body weight) followed by exsanguinations. Backfat and longissimus dorsi (LD) muscle were isolated aseptically. The porcine pre-adipocytes were obtained through the “ceiling culture” method, isolated according to our previous report^21^. The backfat and LD muscle were washed and minced into approximately 1 mm^3^ slices, then digested with 0.1% (w/v) Type I collagenase (Life Technologies, USA). The tubes were incubated for 1 h at 37 °C with gentle shaking. After digestion, the cell suspension was filtrated through a 300-mm screen cloth to remove undigested tissue debris and then centrifuged at 190 g for 10 min. The supernatant containing mature adipocytes was aspirated to new centrifuge tubes with DMEM and then centrifuged at 190 g for 10 min. The steps above were repeated twice to obtain pure mature adipocytes in the top layer of the tube. The fatty layer was transferred to a 25 cm^2^ cell culture flask, which was fully filled with DMEM + 10% fetal bovine serum (FBS), inverted the flask in order to allow the floating mature adipocytes to adhere to the top inner surface, and incubated in a 37 °C incubator with 5% CO_2_ and 95% O_2_. After a 12-h incubation period, the cell media were transferred to new flasks, which were again entirely filled with DMEM + 10% FBS. This purification step was repeated twice in order to remove contaminant fibroblasts and obtain pure mature adipocytes. 10 days later, the mature adipocytes lost lipids and dedifferentiated into pre-adipocytes. Then excess cell culture media was removed and the culture flask was turned right side up. The medium was changed every 3 days until confluence was achieved.

### Adipocytes differentiation

After pre-adipocytes reaching confluence, cells were induced to differentiate with the differentiation medium (DMEM, 10% FBS, 0.25 nM dexamethasone, 0.1 mM 3-isobutyl-1-methylxanthine, and 5 μg/mL insulin) for 3 days, then the medium was replaced with culture medium for another 9 days. All medium was refreshed every 3 days.

### ACTH experiment

Twelve Xinhuai sows were randomly divided into two groups. 1 U/kg adrenocorticotropic hormone (Sigma, USA) or equivalent volume saline was injected via cervical vein per day for 9 days. Then the levels of serum cortisol were detected, and backfat and LD muscle samples were harvested. Isolated the IMAT with Ophthalmic forceps in the LD muscle.

### Muscle conditioned medium

Muscle tissue was isolated from the longissimus dorsi of newborn Erhualian pigs. Ten grams of muscle tissue was minced into 1 mm^3^ pieces in Dulbecco’s Modified Eagle’s (DMEM) medium, transferred to a 25 cm^2^ cell culture flask, and incubated in DMEM containing 10% fetal bovine serum (FBS) at 37 °C in a 5% CO_2_ humidified atmosphere for 12 h. The culture medium was aspirated and filtered through a 300 mm screen cloth to remove the tissue debris. Muscle conditioned medium was prepared by mixing culture medium in DMEM containing 10% FBS at a ratio of 1:2.

### Cell proliferation assay

#### Cell counting

Cell number was counted by the automated cell counter (Invitrogen, USA). Cell viability was determined using the Cell Counting Kit-8 (CCK-8) (Vazyme, Jiangsu, China) assay.

#### Cell viability assay

CCK-8 contains WST-8 which can be deoxidized to the hydrosoluble formazan dye by mitochondrial dehydrogenase in living cells. Cells were treated with 10 μl CCK-8 solutions and incubated at 37 °C for 4 hours. The absorbance was measured using an automated microplate reader (Bio-Rad, Japan) at 450 nm. Results were expressed as percentages of the controls, which were arbitrarily assigned with 100% viability.

#### EdU assay

Adipocytes were cultured with fresh growth medium containing EdU (final concentration,10 mM) for 2 hours. EdU staining was conducted using Cell-Light™ EdU Apollo®488. *In Vitro* Imaging Kit (RiboBio, Guangzhou, China) according to the manufacturer’s protocol. The EdU-labeled cells were imaged with confocal microscope.

### Cell differentiation assay

#### Oil red O staining

Mature adipocytes were washed 3 times with PBS, and then fixed with 10% formalin for 15 min. After fixation, the cells were washed with PBS 3 times and stained with oil red O for 20 min. Subsequently, the cells were washed with 60% isopropanol for 20 s, and then imaged with inverted microscope.

#### Triglyceride assay

The intra-cellular triglyceride was assayed using a triglyceride assay kit (Applygen Technologies Inc) according to the manufacture’s instruction.

### RT-qPCR

Total RNA was isolated from cells or tissues using TRIzol reagent (Invitrogen, Shanghai, China). cDNA for was synthesized using ProtoScript M-MuLV (NEB,USA). The DNA standards were performed using pspt18 vector (Promega, USA) as previously described^40^, and primer sequences were listed in Supplementary Table 1. Real-time PCR was performed using SYBR Premix Ex Taq^TM^ (TaKaRa, Dalian, China) on ABI StepOne Plus™ Real-Time PCR System (Applied Biosystems, USA). Relative expression was analyzed by using 2^−ΔΔCt^ and are referred to the control treatment using *RPLP0* as a reference gene. The primer sequences were listed in Supplementary Table 2.

### Western blot

Total protein extracts with RIPA lysis buffer (Beyotime Biotechnology, Jiangsu, China) and protein quantities were measured by the BCA Protein Assay kit (Beyotime Biotechnology, Jiangsu, China). Antibodies against GRα (sc-393232, Santa Cruz, USA) and tubulin (sc-5274, Santa Cruz, USA) was used in Western blot analysis. Images were captured with VersaDoc 4000MP system (Bio-Rad).

### Apoptosis and Cell Cycle Assay

After adipocytes were harvested and washed, apoptosis of adipocytes was detected with Annexin V-FITC/PI Apoptosis Detection Kit (Vazyme, Jiangsu, China) according to the manufacturer’s instructions. Cell cycle were detected with Cell Cycle Assay Kit (Vazyme, Jiangsu, China). The Cells were sorted by fluorescence-activated cell sorting using a Beckman Coulter instrument (Becton Dickinson, Franklin, NJ, USA). The early apoptosis rate was calculated as AnnexinV (+) PI (−) cells/total cell × 100%, and the late apoptosis rate was calculated as Annexin V (+) PI (+) cells/total cell × 100%, and the necrosis rate was calculated as Annexin V (−) PI (+) cells/total cell × 100%.

### ELISA

The commercial ELISA kits (R&D Systems, USA) was used to measure the cortisol concentration in porcine serum according to the manufacturer’s instruction. The porcine MSTN ELISA Kit (Elabscience, Wuhan, China) was used to determine the concentration of MSTN in MCM and serum according to the manufacturer’s instruction.

### Methylation analysis

#### Bisulfite sequencing

Genomic DNA was extracted using the phenol-chloroform method from SA and IMA pre-adipocytes. Bisulfite treatment was performed according to the manufacturer’s instruction of EZ DNA Methylation-Gold Kit^TM^ (Zymo Research, USA). Primers (BSP- Promoter 1 C, BSP- Promoter 1 H) for the amplification of the GR promoter region were designed by Methyl Primer Express v1.0. The PCR products were cloned in the pEASY-T3 Cloning Vector (Transgen Biotech, Beijing, China), then positive clones were sequenced.

### Methylated DNA immunoprecipitation

This method was carried out followed the previously description^64^. Briefly speaking, 40 μg of DNA was sheared between 200~500 bp by sonication, then immunoprecipitated by 5 μl 5-mC monoclonal antibody (Diagenode, Belgian). Ct values were determined by RT-qPCR, and the Methylation Ratio was calculated by the formula Methylation Ratio (IP/Input) = 2^−(Ct[IP]−Ct[Input])^ × 100%.

### Statistical analysis

At least three biological replicates were used for each analysis. Statistically significant differences were determined using paired t-test or one-way ANOVA of variance using SPSS 18.0 software (SPSS Inc., USA). P-value of 0.05 was considered significant. All values are presented as the mean ± SEM.

## Electronic supplementary material

Dataset 1
