# Supplementary material for: Muscle-specific downregulation of GR levels inhibits adipogenesis in porcine intramuscular adipocyte tissue
Source: Sci Rep. 2017 Mar 30;7:510. doi: 10.1038/s41598-017-00615-9 (PMC5428816; doi:10.1038/s41598-017-00615-9)
Supplement: Supplementary file 1 — Dataset 1 [file 41598_2017_615_MOESM1_ESM.docx]

# SUPPLEMENTARY INFORMATION

# Muscle-specific downregulation of GR levels inhibits adipogenesis in porcine intramuscular adipocyte tissue

Weiwei Chu^a,b,#^, Wei Wei^a,#^, Haiyin Han^a^, Ying Gao^c^, Kaiqing Liu^a^, Ye Tian^a^, Zaohang Jiang^a^, Lifan Zhang^a^, Jie Chen^a, *^

^a^ College of Animal Science and Technology, Nanjing Agricultural University, Nanjing 210095, PR China

^b^ Precision Medicine and Healthcare, Tsinghua-Berkeley Shenzhen Institute, Shenzhen 518055, PR China

^c^ College of Veterinary Medicine, Nanjing Agricultural University, Nanjing 210095, PR China

* Corresponding author at: College of Animal Science and Technology, Nanjing Agricultural University, Nanjing 210095, PR China.

^#^ These authors contributed equally to this work as co-first authors

*E-mail address of corresponding author:* [jiechen@njau.edu.cn](mailto:jiechen@njau.edu.cn) (J. Chen)


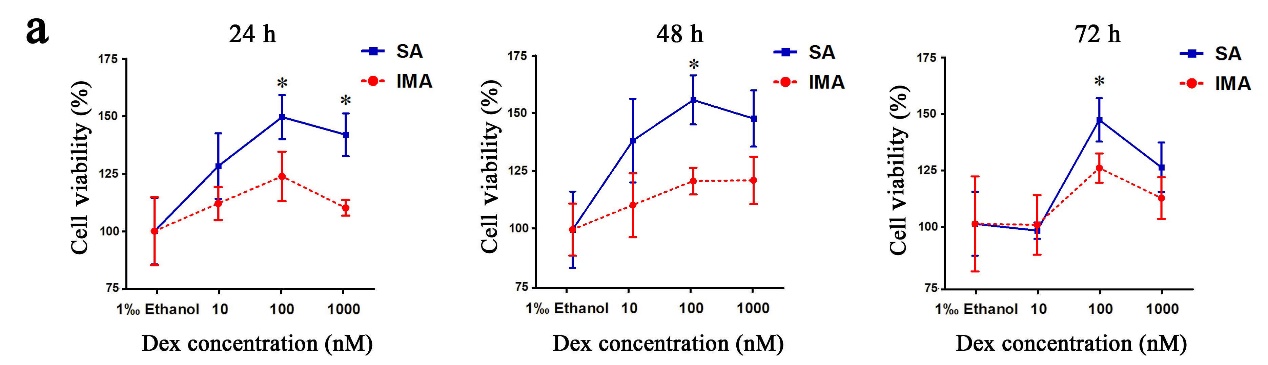


**Figure 1. The DEX induced cell viability is lower in IMA than SA pre-adipocytes.** The porcine SA and IMA pre-adipocytes were cultured with growth media supplemented with 1‰ ethanol or DEX of different concentrations in 96-well culture plates for 3 days, and cell viability was determined by CCK8. Data are shown as the mean ± SEM, n = 8 per group, * *P* < 0.05.


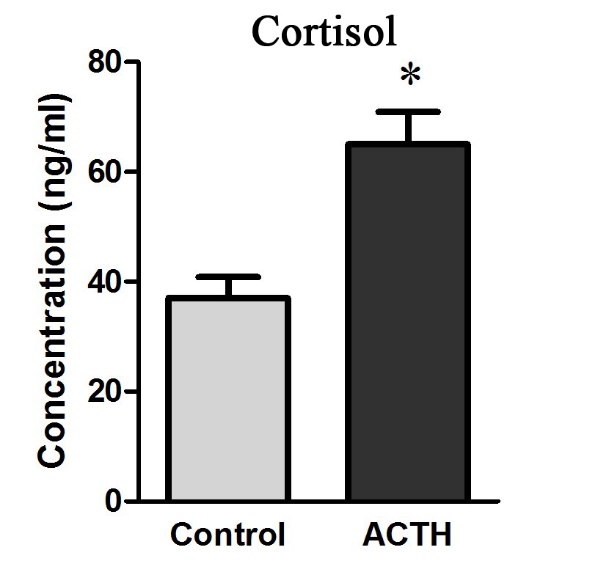


**Figure 2. Concentration of plasma cortisol.** 12 sows were picked and injected 1 U/kg ACTH or equivalent volume saline intravenously per day for a total 9 days, then the plasma cortisol was detected. Data are shown as the mean ± SEM, n =6, * *P* < 0.05.





**Figure 3. The IMAT is less sensitivities to endogenous GCs than SAT.** The sows injected with 1 U/kg ACTH (n = 6) or equivalent volume saline (n = 6) intravenously per day for a total 9 days, then the fold changes of mRNA expression in SAT and IMAT were detected. * means significant differences between control and ACTH groups, **P* < 0.05, ***P*< 0.01.


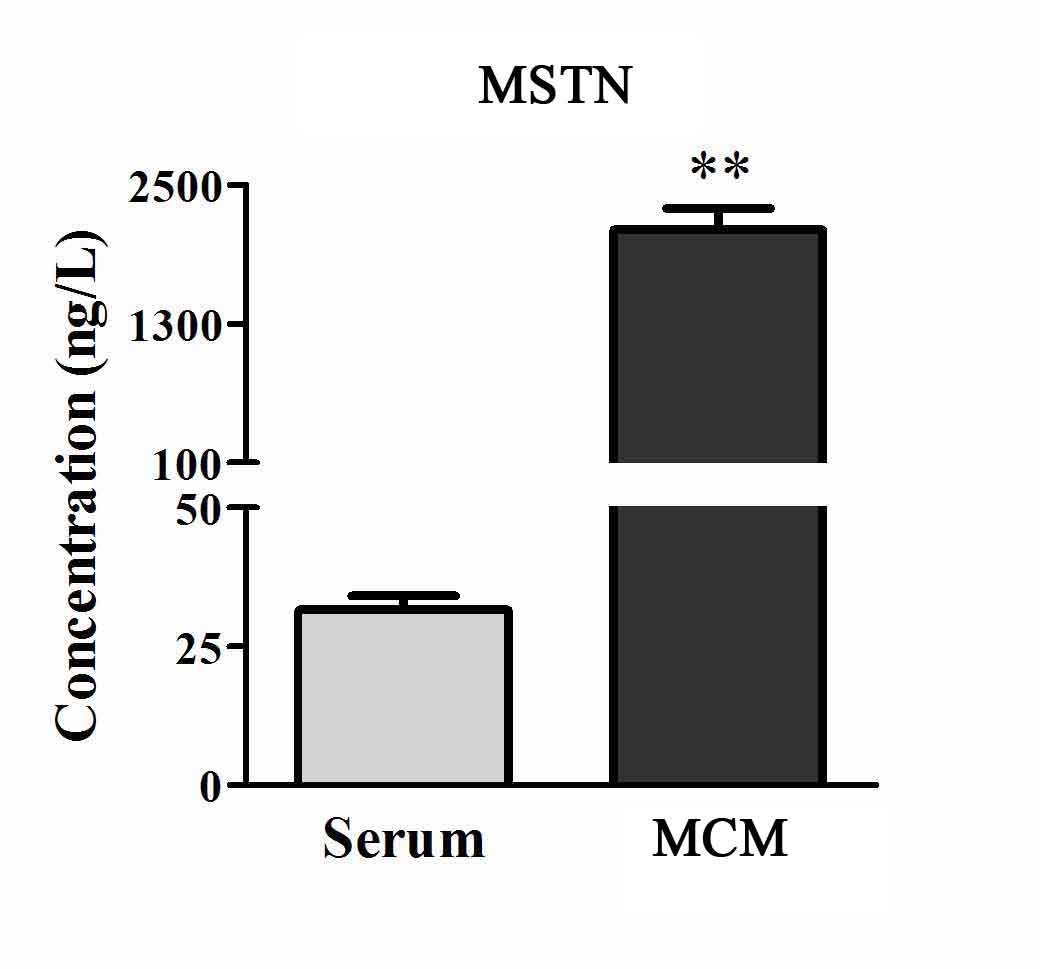


**Figure 4.** Concentration of MSTN in serum and muscle conditioned medium. Data are shown as the mean ± SEM, n = 6, * * *P* < 0.01.

Tab.1 The primer sequences for the DNA standards of GR variants

| **Gene** | **Forward Primer （5' to 3'）** | **Reverse Primer （5' to 3'）** |
| --- | --- | --- |
| pspt18-totalGR | ACGGAATTCCCAAGGAATCGCTGACCC | ACGAAGCTTATTGCTTCCTGAGCCTTTTG |
| pspt18-GRα | ACGGAATTCGCCATTGTCAAGAGGGAAGGA | ACGAAGCTTGGGGAAAATACACCAACGAAAG |
| pspt18-GRβ | ACGGAATTCGCTTCAGGTGTCTTACG | ACGAAGCTTAATTACATGATACCAATCAG |
| pspt18-1A | ACGGAATTCTACCCTCCTGGGGCTTTTATCG | ACGAAGCTTTGCTGGGGATTTCTTCTCTGCTG |
| pspt18-1B | ACGGAATTCATGCGGGGGAGGGGGACC | ACGAAGCTTATTGCTTCCTGAGCCTTTTG |
| pspt18-1C | ACGGAATTCCCTGCTTTCACACGCTAA | ACGAAGCTTACGCTGCTGGGGATTTC |
| pspt18-1D | ACGGAATTCATCGTAATATGTGCGGCC | ACGAAGCTTATTGCTTCCTGAGCCTTTTG |
| pspt18-1E | ACGGAATTCCATTTGGCGTGCAACTTCCTTC | ACGAAGCTTGCAGCGGAGGCTGAACTCTTG |
| pspt18-1F | ACGGAATTCGCGGCGAAGAGAAACTAGAG | ACGAAGCTTATTGCTTCCTGAGCCTTTTG |
| pspt18-1G | ACGGAATTCCAGTTGCCGAGCGTCGCC | ACGAAGCTTCTTGGGGTCCATCAGTGGGTATCA |
| pspt18-1H | ACGGAATTCCTGGTGGAAGTGGGCGTGTC | ACGAAGCTTTTCCTCCCCTCAGGCTTTTAT |
| pspt18-1J | ACGGAATTCAACTTGGATGCGGGCC | ACGAAGCTTATTGCTTCCTGAGCCTTTTG |

Tab.2 The primer sequences for RT-qPCR

| **Gene** | **Forward Primer （5' to 3'）** | **Reverse Primer （5' to 3'）** |
| --- | --- | --- |
| FASN | AGG CGT GCT CCG TCT GCT T | GAG TGG CGA ATG GAA AGG |
| PPARγ | TGG AAC CCC GAG GCT TTA T | GAA CGG GCG AAA GAA CA |
| HSL | GCC CGA GAC GAG ATT AGC A | ATG AAG GGA TTC TTG ACG ATG |
| ATGL | GAA CAT CTC GTT CGC GGG T | ACA CCT CGA TGA TGC TGG C |
| Total GR | CCAAGGAATCGCTGACCC | ATTGCTTCCTGAGCCTTTTG |
| GRα | GCCATTGTCAAGAGGGAAGGA | GGGGAAAATACACCAACGAAAG |
| GRβ | GCTTCAGGTGTCTTACG | AATTACATGATACCAATCAG |
| Exon 1A | TACCCTCCTGGGGCTTTTATCG | TGCTGGGGATTTCTTCTCTGCTG |
| Exon 1B | ATGCGGGGGAGGGGGACC | ATTGCTTCCTGAGCCTTTTG |
| Exon 1C | CCTGCTTTCACACGCTAA | ACGCTGCTGGGGATTTC |
| Exon 1D | ATCGTAATATGTGCGGCC | ATTGCTTCCTGAGCCTTTTG |
| Exon 1E | CATTTGGCGTGCAACTTCCTTC | GCAGCGGAGGCTGAACTCTTG |
| Exon 1F | GCGGCGAAGAGAAACTAGAG | ATTGCTTCCTGAGCCTTTTG |
| Exon 1G | CAGTTGCCGAGCGTCGCC | CTTGGGGTCCATCAGTGGGTATCA |
| Exon 1H | CTGGTGGAAGTGGGCGTGTC | TTCCTCCCCTCAGGCTTTTAT |
| Exon 1J | AACTTGGATGCGGGCC | ATTGCTTCCTGAGCCTTTTG |
| RPLP0 | TCCAGGCTT TAGGCA TCACC | GGCTCCCACTTTGTCTCCAG |
| BSP-Promoter 1C | GGAGGGTGTATTTTGTAAGTAA | AAATTACAAAACAAAACCCAC |
| BSP-Promoter 1H | TYGTYGGAGTTTGTAAATTTT | CAAACCTATTAAATTCTTCCCC |
